# Gated Volumetric-Modulated Arc Therapy vs. Tumor-Tracking CyberKnife Radiotherapy as Stereotactic Body Radiotherapy for Hepatocellular Carcinoma: A Dosimetric Comparison Study Focused on the Impact of Respiratory Motion Managements

**DOI:** 10.1371/journal.pone.0166927

**Published:** 2016-11-22

**Authors:** KyoungJun Yoon, Jungwon Kwak, Byungchul Cho, Jin-hong Park, Sang Min Yoon, Sang-wook Lee, Jong Hoon Kim

**Affiliations:** Department of Radiation Oncology, Asan Medical Center, University of Ulsan College of Medicine, Seoul, Republic of Korea; Yonsei University College of Medicine, REPUBLIC OF KOREA

## Abstract

**Purpose:**

To assess the potential dosimetric benefits associated with the CyberKnife (CK) tumor tracking capability, wherein an extra margin for respiratory tumor motion is not required, when compared to respiratory-gated volumetric-modulated arc therapy (VMAT) for hepatocellular carcinoma (HCC).

**Methods:**

Twenty-nine HCC patients previously treated with double-arc VMAT were enrolled. In each VMAT plan, the individual internal target volume (ITV) margin around the tumor was determined by measuring its motion over 30–70% of respiratory phases using four-dimensional computed tomography, followed by a 5-mm isotropic margin for the planning target volume (PTV). For each VMAT plan, two CK plans were generated using the original (CK_original_, ITV included) and modified PTVs (CK_modified_, ITV excluded) for comparison. In each case, the CK_original_ and CK_modified_ plans were compared to the original VMAT plan in terms of the dosimetric parameters including the conformity index (CI), PTV coverage (CO), organs at risk (OAR) doses, and normal liver tissue sparing.

**Results:**

The original PTVs with median 24 cc (range, 9–65 cc) were significantly reduced to median 12 cc (range, 5–41 cc) in the CK_modified_ plans. Statistically significant differences in plan qualities were observed between the VMAT and the CK plans: mean CI, 1.05 in VMAT vs. 1.17 in both CK plans (*p* < 0.001); and mean CO, 93.0% in VMAT vs. 96.6% in CK_original_ and 96.9% in CK_modified_ (*p* < 0.001). The average volume of normal liver tissue receiving > 15 Gy was significantly decreased in the CK_modified_ plan, as compared to that in the VMAT and CK_original_ plans, by 1.75- and 1.61-fold, respectively.

**Conclusions:**

The tumor tracking capability of the CK system can significantly decrease the volume of normal liver tissue receiving > 15 Gy, while maintaining high precision in target localization, conformity, tumor coverage, and dose sparing of the OAR. Therefore, it can be a valuable SBRT option, particularly for HCC patients with poor liver function.

## Introduction

Hepatocellular carcinoma (HCC) is one of the most common malignancies and the third most common cause of cancer-related deaths worldwide [1]. Surgery is the standard treatment for HCC, with 5-year survival rates of 50–70%; however, surgery is only suitable for < 30% of HCC patients at diagnosis [2–5]. Other modalities such as radiofrequency ablation or percutaneous ethanol injection have also been used as curative treatment options [6, 7]; however, these options are limited in cases where the HCC lesions are positioned at the top of the dome, at deep locations, and/or close to the main blood vessels (or bile duct). Therefore, non-invasive local therapeutic modalities such as radiotherapy (RT) are the choices of treatment in these clinical settings.

Recent advances in RT technology not only enable highly focused dose delivery using intensity-modulated radiotherapy (IMRT) and volumetric-modulated arc therapy (VMAT), but also facilitate precise tumor localization with image guidance during treatment. With these techniques, stereotactic body radiotherapy (SBRT) can deliver a highly focused ablative dose to the tumor, and accordingly achieve effective tumor control while minimizing the radiation-induced toxicity to normal tissue. Several studies have reported that an SBRT dose of 24–60 Gy administrated in 3–6 fractions is safe and efficacious for the treatment of HCC [8–12]. Considering the radiobiologically parallel architecture of liver tissue, preserving the ‘critical volume’ of normal liver tissue is essential for preventing radiation-induced liver injury by SBRT [10, 13, 14]. Schefter et al. suggested 700 cc of the normal liver volume receiving < 15 Gy in 3 fractions or less as the critical volume of liver [15].

In this regard, respiratory induced tumor motion is a challenge in SBRT for HCC, especially when tumor motion or its volume is large. When radiation is delivered during free breathing, tumor can move up to 3 cm and therefore a significant amount of geometric margin that accounts for the tumor location over the entire breathing cycle is needed. Minimizing the irradiation of normal liver tissue without missing the tumor requires efficient motion management strategies for the success of SBRT. As reducing the component of the planning target volume (PTV) that accounts for respiratory motion can spare normal liver tissue, various respiratory motion management strategies have been adopted thus far, including active breath-holding, abdominal compression, and respiratory gating and tracking. However, active breath-holding is not suitable for ~40% of the patients receiving SBRT [16], abdominal compression reduces liver motion by an average of only 2.3 mm [17], and gating requires continuous real-time monitoring and a larger online workload owing to 30–50% duty cycle [18]. Recent approaches involving mean breathing tumor localization and treating breathing motion as a random error appear promising for reducing the tumor margin to one-third of the breathing amplitude; however, these measures are not commonly used due to the difficulties in establishing a reliable mean breathing position using four-dimensional computed tomography (4D-CT) [19].

All the above-mentioned strategies for respiratory motion management can be used with modern linac-based SBRT, and can effectively reduce the margin associated with respiratory tumor motion to within ~5 mm. Nevertheless, real-time tumor tracking is ideal for SBRT because it prevents extra irradiation by accounting for the respiratory tumor motion. However, it is only possible with the CyberKnife (CK) Synchrony, a real-time tumor motion tracking system (Accuray Inc., Sunnyvale, CA) [20–22].

SBRT can be performed either by using a linac-based VMAT that simultaneously incorporates gantry rotation, dose rate modulation, and continuous multi-leaf collimator (MLC) motion with or without respiratory gating (including the ITV margin), or by using a non-isocentric robot-based CK Synchrony system for respiratory real-time tumor motion tracking (excluding the ITV margin). Although two modalities employ markedly different beam delivery systems, including isocentric vs. non-isocentric, MLC vs. cone-type collimator, and respiratory gating vs. respiratory tracking, direct comparison studies of dosimetric characteristics in SBRT between two modalities, particularly focusing on the effect of the ITV margin, are rare.

A recent dosimetric comparison study between VMAT and CK in liver SBRT by Paik et al. [23] reported that CK plans showed better dose conformity, particularly in small-sized tumors whereas VMAT showed better low dose distributions in the normal liver and a shorter delivery time. In the study, the GTV was contoured on slow CT to ensure that the PTV encompasses the entire respiratory induced tumor motion; thus, no motion management was applied at all. However, the AAPM TG 76 [24] recommends active motion management for respiratory motion > 5 mm, which is even more important in SBRT. As the CK plan used the same PTV volume without considering tumor tracking feature of CK, the CK plan in the Paik et al study was not a realistic one. Instead, Paik and colleagues focused on the dosimetric consequences due to the difference in the beam delivery system, which is similar to the CK_original_ plan (will be explained later) in the present study. Hence, their results reflected the variation caused by the differences between isocentric vs. non-isocentric, MLC vs. cone-based beam shaping, and the inverse planning optimization algorithm.

In the present study we aimed not only to investigate the dosimetric characteristics of these two SBRT techniques by comparison of their plans using the identical treatment planning CT images and contour sets, but also to assess the impact of respiratory motion managements on plan quality along with the potential dosimetric benefits of the CK tumor tracking system for which ITV is not necessary, in comparison with those offered by the linac-based respiratory-gated treatment system.

## Materials and Methods

### 1. Patient selection and 4D-CT simulation

The Institutional Review Board of Asan Medical Center approved this study, and the requirement for informed consent was waived because of the retrospective nature of the analysis. In total, 121 patients underwent SBRT for HCC with the respiratory-gated VMAT technique at our institution between October 2014 and February 2015. Among these patients, 29 (median age, 66 years; 8 women and 21 men) with a representation of the tumor location were retrospectively selected and analyzed.

The detailed procedure for liver SBRT at our institution has been described in previous reports [25, 26]. If appropriate surrogates for tumor localization, including surgical clips or compact iodized oil remaining from previous treatments were available, they were used for tumor localization during treatment (n = 20). In other cases, three fiducial markers (CIVCO medical solutions, Kalona, IA) were inserted into the liver parenchyma around the tumor at least one week prior to 4D-CT simulation (n = 9).

Four-dimensional CT scans were acquired during free breathing using a GE LightSpeed RT16 CT simulator. A real-time position management system (RPM, Varian medical systems, Palo Alto, CA) was used to record the patients’ respiratory data. The 4D-CT images synchronized with the respiratory data were sorted into 10 CT series based on the respiratory phase. All 10 CT data were then transferred to a Varian Eclipse treatment planning system for contouring and planning.

The gross tumor volume (GTV) was delineated at the end-exhale (50% or 60%) phase CT image referring to the contrast enhanced CT and/or magnetic resonance imaging (MRI). The clinical target volume (CTV) was not defined. Each directional tumor motion from the end-exhale position was measured for the respiratory gating window of mostly 30–70% from the 10-phase 4D-CT images. The measured non-isotropic tumor motion in every direction was added to the GTV to define the internal target volume (ITV) for gated RT. The PTV margin of 5 mm was expanded in all directions from the ITV to account for the uncertainties associated with target definitions, inter-, and intra-fractional tumor localizations. The organs at risk (OAR) such as normal liver, duodenum, spinal cord, kidney, and stomach were delineated.

Using the delineated target and OAR volumes, original VMAT plans were generated on the end-exhale phase CT image using the Eclipse TPS, as described in the following sections.

For dosimetric comparison, the original contour sets for the VMAT plans in Eclipse TPS were transferred to CK MultiPlan TPS (version 4.5) to ensure identical images and contour sets, and two CK plans were retrospectively produced either with or without ITV, as described in the following sections.

The PTV contour sets of the three plans were defined as follows: (1) VMAT_PTV = GTV + ITV (gating phase window, 30–70%) + 5 mm for the VMAT plans, (2) CK_original__PTV = VMAT_PTV for the CK_original_ plans, and (3) CK_modified__PTV = GTV + 5 mm for the CK_modified_ plans.

Patient characteristics with different PTVs are summarized in Table 1. The average respiratory tumor motion during free breathing was 14.5 ± 5.7 mm, which was reduced to 5.0 ± 2.3 mm with gating. Accordingly, the average volume of the PTVs of the original VMAT and CK_original_ plans was 27.1 ± 15.0 cc, which were reduced to 15.6 ± 10.0 cc with the CK_modified_ plans. The average volume of the GTVs was 5.1 ± 5.0 cc with a diameter range of 1–4 cm, and the mean volume of the normal liver tissue excluding the GTV was 1163.7 ± 199.7 cc.

**Table 1:** Patient characteristics.

Characteristic	^a^Value (%)
Tumor motion (mm)	
Full-motion	14.5 ± 5.7
1–5 mm	1 (3.4)
6–10 mm	7 (24.1)
11–15 mm	9 (31)
16–20 mm	8 (27.6)
21–30 mm	4 (13.8)
Gating-motion (ITV)	5.0 ± 2.3
1–5 mm	18 (62.1)
6–10 mm	11 (37.9)
Tumor location	
Central	10 (34.4)
Periphery	14 (48.3)
Dome	5 (17.3)
Marker type	
Surgical clip	2 (6.9)
Lipiodol	18 (62.1)
Gold seed	9 (31.0)
GTV (cc)	5.1 ± 5.0
Diameter of GTV (mm)	2.0 ± 0.6
10–20 mm	19 (65.5)
21–30 mm	7 (24.1)
31–40 mm	3 (10.3)
PTV (cc)	
VMAT and CK_original_	27.1 ± 15.0
CK_modified_	15.6 ± 10.0
Diameter of PTV (mm)	
VMAT and CK_original_	3.6 ± 10.6
20–30 mm	6 (20.7)
31–40 mm	17 (58.6)
41–50 mm	6 (20.7)
CK_modified_	3.0±0.6
20–30 mm	17 (58.6)
31–40 mm	9 (31.0)
41–50 mm	3 (10.3)
Normal liver volume (nlV, exclude GTV) (cc)	1163.7 ± 199.7

^a^Values are presented as mean ± standard deviation or number of patients.

### 2. Treated VMAT plans

In the original VMAT plans, a total dose of 45 Gy in 3 fractions was prescribed for all patients with the following planning objectives: prescription dose to cover at least 95% of PTV, conformity index (CI) < 1.2, and appropriate dose sparing of the OARs. The normal tissue dose constraints used at our institution for liver SBRT in 3 fractions are presented in Table 2 [26]. The Eclipse analytical anisotropic algorithm was used for dose calculation with a grid size of 1.25 mm in the VMAT plans. Using the Eclipse planning system, inverse planning for each patient was performed with a 10 MV flattening-filter-free beam of a Varian TrueBeam accelerator equipped with a high-definition 120 MLC, and 2 full arcs (clockwise rotation from 181–179° with the collimator at 30°, and counterclockwise rotation from 179–181° with the collimator at 330°). The mean isodose line prescribed to 45 Gy was 90.6% (range, 87–92%) of the maximum dose.

**Table 2:** Normal tissue tolerance for HCC SBRT in 3 fractions.

Organ at Risk	Constraints
Normal Liver	V_d<15Gy_ > 700 cc
Normal Liver	D_mean_ < 13 Gy
Esophagus	D_2cc_ < 21 Gy
Large bowel	D_2cc_ < 21 Gy
Stomach	D_2cc_ < 18 Gy
Duodenum	D_2cc_ < 18 Gy
Spinal cord	D_max_ < 18 Gy

### 3. Retrospective CK plans

For each of the 29 VMAT plans, two CK plans, either including or excluding ITV, were retrospectively generated using identical CT images and contour sets with the original VMAT plan. The CK_original_ plans that included the ITV were generated to compare the plan quality between VMAT and CK with the same tumor volumes, whereas CK_modified_ plans that excluded the ITV were generated to assess the dosimetric benefits associated with the tumor tracking capability of the CK system. Both CK plans were generated using the ray-tracing dose calculation algorithm. For the MultiPlan planning system, sequential optimization planning was performed for each patient using a 6 MV flattening-filter-free beam generated by the CK radiosurgery system (version 9.5). One to three fixed collimators 30–70% of the tumor diameters were selected depending on the tumor size and shape. Five dose-limiting auto-shells were utilized to improve both dose conformity and compactness at each dose level as follows: at the prescription dose (PD) level for dose conformity, intermediate dose level (50% of the PD) for rapid dose fall-off, and low dose levels (10–30% of the PD) for low dose spillage. Depending on the tumor size, dose-limiting auto-shells were created around the PTV with a median gap of 2 mm (range, 1–3 mm) for the first shell, 8 mm (range, 5.5–10 mm) for the second shell, 15 mm (range, 15–25 mm) for the third shell, 25 mm (range, 24–35 mm) for the fourth shell, and 40 mm (range, 36–50 mm) for the fifth shell. The mean isodose line prescribed to 45 Gy was 82.8% (range, 80–87%) of the maximum dose in CK_original_, and 84.1% (range, 80–89%) of the maximum dose in CK_modified_.

### 4. Comparison of dosimetric parameters and statistical analysis

The dosimetric characteristics of the datasets for each plan (VMAT plan, CK_original_ plan, and CK_modified_ plan) were compared using the following parameters: 1) the minimum, mean, and maximum doses in the target volume; 2) CI, defined as the ratio of the ‘PIV/TV_PIV_’, where the TV_PIV_ is the target volume (TV) in the prescription isodose volume (PIV); 3) tumor coverage (CO), defined as the ratio of the ‘TV_PIV_/TV’; 4) homogeneity index (HI), defined as the ratio of ‘maximum dose/PD’; 5) dose gradients (or intermediate- and low-dose spillages) (GI_50%_ and GI_15Gy_), as the ratio of the volume that received 50% of the prescribed dose, and 15 Gy to the PIV [27], respectively; 6) normal liver volume receiving > 15 Gy (nlV_D >15Gy_). In addition, for the analysis of normal liver outside the PIV, the critical normal liver volumes receiving < 5–45 Gy, in increments of 5 Gy (clV_D<5 Gy to 45Gy_) were obtained and normalized to the total normal liver volume, and compared. The dosimetric indices for the three planning groups were analyzed using the repeated measured analysis of variance (ANOVA) test (SPSS Statistics, version 20, IBM, NY). The significance levels were determined by Bonferroni correction for multiple comparisons (*p* ≤ 0.05/3).

## Results

**Fig 1:**
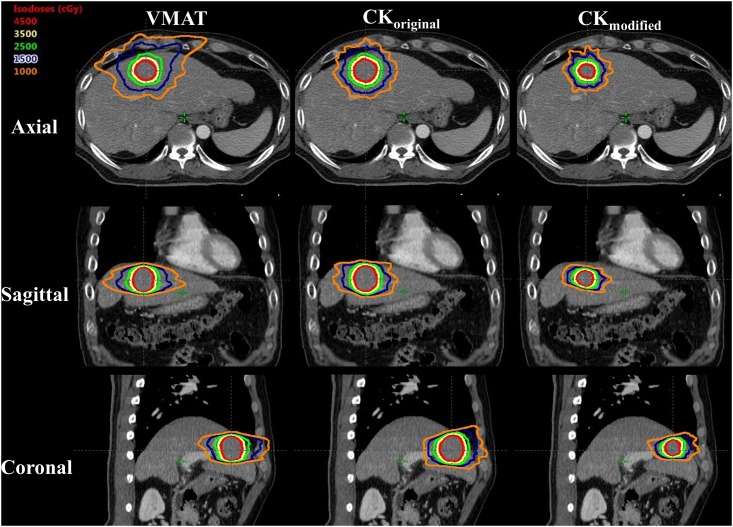
Dose distributions of the (a) VMAT, (b) CK_original_, and (c) CK_modified_ plans (Case #2); the dose of 45 Gy in 3 fractions is prescribed to the red isodose line that covers the PTV. The isodose line was shown in each case, with the colored lines corresponding to the following doses: red = 45 Gy, yellow = 35 Gy, green = 25 Gy, dark blue = 15 Gy, and orange = 10 Gy, respectively. The PTV, CI, and HI were “17.6 cc, 1.02, and 1.09” for VMAT, “17.7 cc, 1.12, and 1.13” for CK_original_, and “5.3 cc, 1.18, and 1.12” for CK_modified_ plans, respectively. The critical normal liver volumes receiving < 15 Gy (clV_D<15Gy_) were 1236.9 cc for VMAT, 1233.6 for CK_original_, and 1294.1 for CK_modified_ plans, respectively.

**Fig 2:**
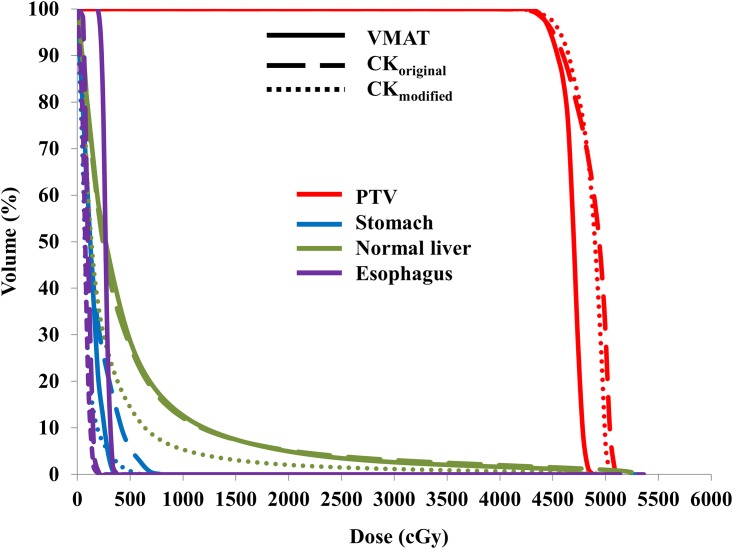
DVHs for the VMAT (solid line), CK_original_ (dashed line), and CK_modified_ plan (dotted line) in Case #2. The PTV (red), stomach (blue), normal liver (green) and esophagus (purple) are shown.

Representative dose distribution and dose-volume histograms (DVHs) for the VMAT, CK_original_, and CK_modified_ plans are depicted in Figs 1 and 2, respectively. As expected, the CK_modified_ plan without ITV margins resulted in a smaller PTV and thus a smaller dose to the normal liver tissue, as compared to the VMAT and CK_original_ plans.

The treatment planning parameter and dosimetric indices for the VMAT, CK_original_, and CK_modified_ plans are summarized in Table 3. All the VMAT tumor volumes in the Eclipse planning system were well reproduced in the MultiPlan CK planning system. The prescription isodose lines reached 95.2% of the maximum dose for VMAT, 82.8% for CK_original_, and 84.1% for CK_modified_. The mean and maximum doses to tumor volumes, and accordingly HI, were significantly higher in CK_original_ and CK_modified_ than in VMAT (*p* < 0.001). No significant differences in the minimum doses were observed between the VMAT and the CK_modified_ plans (*p* = 0.848).

**Table 3:** Summary of the VMAT and CK plan parameters (29 cases).

	1	2	3	Multiple comparison			
	VMAT	CK_original_	CK_modified_	*p*-value^a^			
	Mean ± SD	Mean ± SD	Mean ± SD	Overall	1 vs. 2	1 vs. 3	2 vs. 3
Prescription (%)	95.2 ± 2.5	82.8 ± 2.4	84.1 ± 2.6	< 0.001	< 0.001	< 0.001	0.002
Min dose (Gy)	41.1 ± 2.0	39.8 ± 2.0	41.1 ± 1.4	< 0.001	< 0.001	< 0.848	< 0.001
Mean dose (Gy)	47.3 ± 0.6	50.8 ± 1.3	50.3 ± 1.2	< 0.001	< 0.001	< 0.001	0.037
Max dose (Gy)	49.6 ± 0.8	54.5 ± 1.6	53.6 ± 1.7	< 0.001	< 0.001	< 0.001	0.002
Treatment time	17.4 ± 1.1	62.7 ± 7.5	58.5 ± 7.9	< 0.001	< 0.001	< 0.001	< 0.001
CI	1.05 ± 0.05	1.17 ± 0.07	1.17 ± 0.05	< 0.001	< 0.001	< 0.001	0.930
CO (%)	93.0 ± 3.9	96.6 ± 3.0	96.9 ± 2.5	< 0.001	< 0.001	< 0.001	0.389
HI	1.10 ± 0.02	1.21 ± 0.03	1.19 ± 0.04	< 0.001	< 0.001	< 0.001	0.001
GI_50%_^b^	3.83 ± 0.56	3.42 ± 0.35	3.62 ± 0.40	< 0.001	< 0.001	< 0.005	< 0.001
GI_15Gy_^c^	8.07 ± 1.54	6.22 ± 0.75	6.64 ± 0.94	< 0.001	< 0.001	< 0.001	< 0.001
nlV_D>15Gy_^d^ (cc)	128.0 ± 71.4	117.6 ± 58.0	73.1 ± 42.6	< 0.001	0.089	< 0.001	< 0.001

SD = standard deviation; HI = homogeneity index; CI = conformity index; CO = coverage; GI = gradient index,

^a^Repeated measures ANOVA test

^b^GI_50%_ defined as the ratio of the volume receiving > 50% of the prescription dose to the prescription isodose volume

^c^GI_15Gy_ defined as the ratio of the volume receiving > 15 Gy to the prescription isodose volume.

^d^nlV_D>15Gy_, normal liver volume (excluding the GTV) receiving > 15 Gy.

**Fig 3:**
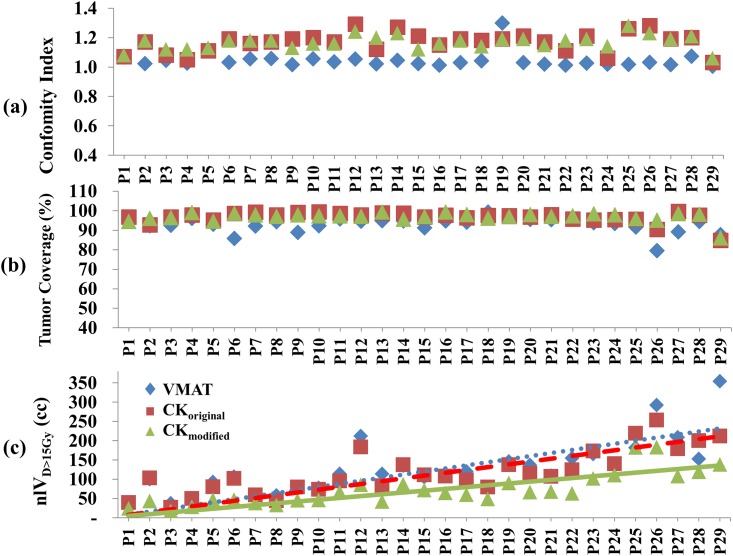
Dosimetric indices of the VMAT (◆), CK_original_ (■), and CK_modified_ (▲) plans for all cases (n = 29 per group): (a) Conformity index; (b) Tumor coverage (%); (c) Normal liver volume receiving > 15 Gy (nlV_D>15Gy_).

Although the planning objective for dose coverage was that the prescription dose should cover at least 95% of the PTV, the final plan approved for treatment was slightly compromised, due to the need for considering various clinical factors such as surrounding OAR dose constraints and patient performance. Therefore, the PTV coverage of VMAT plans showed some variation (93.0 ± 3.9%), as shown in Table 3. To maintain the original plan as it was delivered, we did not attempt to further normalize the plans so that the 95% of the PTV should receive the prescription dose. In fact, we found that the CK plans (CI, 1.17; CO, 97%) showed slightly better coverage than VMAT plan (CI, 1.05; CO, 93%) for the cohort in this study, as shown in Fig 3(a) and 3(b).

As a key index for dose sparing of the surrounding normal tissue, the GI_50%_ for CK_original_ (3.42 ± 0.35) and CK_modified_ (3.62 ± 0.40) was smaller than that for VMAT (3.83 ± 0.56). The similar GI_15Gy_ trends with the CK plans demonstrated that they achieved a more rapid dose fall-off around the tumor than the VMAT plans, and consequently saved more critical normal liver tissue at a dose level of 15 Gy. Table 4 summarizes the results for the critical normal liver volume receiving doses < 5–45 Gy. The critical normal liver volumes receiving < 15 Gy, i.e., clV_D <15Gy_ > 700 cc are generally used to evaluate dose-limiting toxicity such as radiation-induced liver disease (RILD). All CK and VMAT plans satisfied the clV_D <15Gy_ > 700 cc constraints. However, the CK_modified_ plans achieved a higher level of clV_D <15Gy_ (1091.2 ± 195.3 cc) than the VMAT (1034.3 ± 205.3 cc, *p* < 0.001) and CK_original_ (1045.7 ± 196. cc, *p* < 0.001) plans. Accordingly, the CK_modified_ plan was better than the VMAT and CK_original_ plans because a high volume of normal liver tissue was spared. In summary, by eliminating the ITV margin of respiratory-gated treatment, the CK_modified_ plans saved approximately 60 cc more critical normal liver volume than the VMAT plans, considering a mean normal liver volume of 1164 cc in our patient cohort.

**Table 4:** Summary of normal liver volume receiving < 5–45 Gy.

	1	2	3	Multiple comparison			
	VMAT	CK_original_	CK_modified_	*p*-value^a^			
	Mean ± SD	Mean ± SD	Mean ± SD	Overall	1 vs. 2	1 vs. 3	2 vs. 3
clV_D<5Gy_^b^	749.2 ± 251.4	667.6 ± 262.1	801.9 ± 258.7	< 0.001	< 0.001	0.002	< 0.001
clV_D<10Gy_	931.6 ± 224.5	961.4 ± 215.5	1033.0 ± 204.5	< 0.001	0.010	< 0.001	< 0.001
clV_D<15Gy_	1034.3 ± 205.3	1045.7 ± 196.9	1091.2 ± 195.3	< 0.001	0.060	< 0.001	< 0.001
clV_D<20Gy_	1087.0 ± 198.0	1084.2 ± 192.7	1115.3 ± 194.7	< 0.001	0.292	< 0.001	< 0.001
clV_D<25Gy_	1112.3 ± 196.3	1104.7 ± 192.5	1128.1 ± 195.2	< 0.001	< 0.001	< 0.001	< 0.001
clV_D<30Gy_	1126.5 ± 196.2	1117.7 ± 193.3	1136.3 ± 195.9	< 0.001	< 0.001	< 0.001	< 0.001
clV_D<35Gy_	1135.2 ± 196.5	1127.4 ± 194.3	1142.5 ± 196.5	< 0.001	< 0.001	< 0.001	< 0.001
clV_D<40Gy_	1141.8 ± 197.0	1135.6 ± 195.5	1147.8 ± 197.3	< 0.001	< 0.001	< 0.001	< 0.001
clV_D<45Gy_	1148.4 ± 197.7	1143.4 ± 197.0	1152.9 ± 198.2	< 0.001	< 0.001	< 0.001	< 0.001

SD = standard deviation, N/A = none applicable

^a^Repeated measures ANOVA test

^b^clV_D<5Gy_ (cc), critical normal liver volume (excluding the GTV) receiving < 5 Gy.

**Fig 4:**
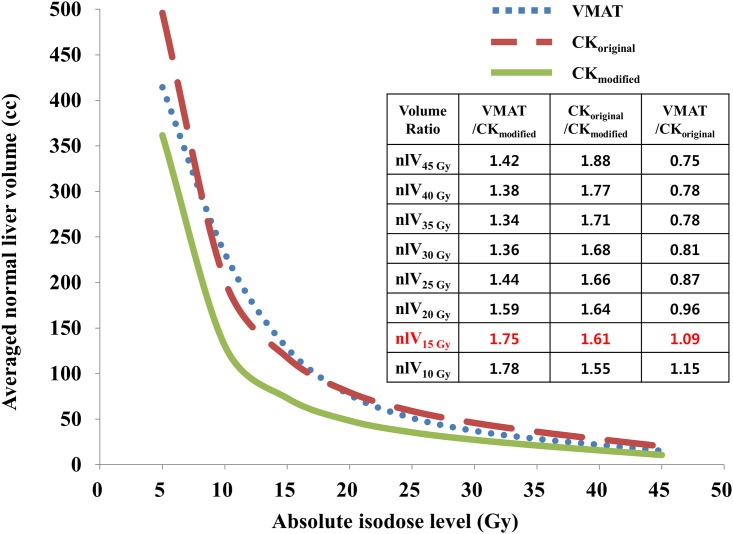
Average DVHs of the normal liver (excluding the GTV) in 29 HCC patients with the VMAT, CK_original_ and CK_modified_ plans. The prescription dose for all plans was 45 Gy in 3 fractions.

As shown in Fig 3(c), the CK_modified_ plan without ITV margins achieved a better plan quality with a smaller volume of normal liver tissue receiving > 15 Gy, as compared to that in the VMAT and CK_original_ plans. Fig 4 shows the relationship between dose and the irradiated normal liver volume. Although all the critical organ doses were well within the tolerated dose-volume limits in all plans, the average normal liver volume receiving > 5–45 Gy was significantly decreased in the CK_modified_ plans as compared to that in the VMAT and CK_original_ plans by 1.39- and 1.50-fold, respectively (*p* < 0.001, each). In particular, the average normal liver volume receiving > 15 Gy was significantly decreased in the CK_modified_ plans compared to that in the VMAT and CK_original_ plans by 1.75- and 1.61-fold, respectively (*p* < 0.001, each).

## Discussion

SBRT for HCC is technically challenging. A significant amount of irradiation margin is required to account for the treatment uncertainties including: 1) tumor delineation errors due to its low contrast in CT simulation images or limited accuracy in image registration with diagnostic MRI; 2) tumor targeting errors due to insufficient contrast for liver masses on in-room cone beam CT and X-ray imaging; and 3) respiratory tumor motion during treatment. In contrast, the risk of developing RILD due to high radio-sensitivity of the normal liver imposes severe restraints on it. Moreover, considering the emerging evidence of the dose-response relationships in HCC, wherein higher doses lead to improved outcomes [28, 29], the narrowest possible safety margin is a prerequisite for maximizing the therapeutic ratio. Consequently, the key factors for successful liver SBRT include 1) capability of generating good plans involving a highly focused dose to the tumor and rapid dose fall-off for dose sparing of critical organs; 2) precise tumor targeting capability either using in-room X-ray or cone beam CT; and 3) efficient respiratory tumor motion management either using gating or tracking.

In the present study, we confirmed that both linac-based VMAT and robotic arm-based CK could provide appropriate plan quality for liver SBRT via the comparison of the VMAT and CK plans, even though their delivery characteristics are quite different. A recent similar study [29] reported that CK plans show greater dose conformity, particularly in small-sized tumors, whereas VMAT plans show good dosimetric distribution of low dose sparing the normal liver and the body. This might be explained by the fact that hundreds of non-isotropic beams of CK can create a highly conformal dose distribution with a sharp dose fall-off, as compared to the isocentric coplanar arc beams of VMAT.

High-energy linear accelerators provide at least dual photon energies such as 6 MV and 10 MV, whereas CK has a single 6 MV flattening-filter free (FFF) beam for compact robot-mounted design. Due to the large beam path length of the abdomen the use of high energy (>10 MV) in liver SBRT can reduce hot spots in the surrounding normal liver tissue and critical organs such as the spinal cord, duodenum, and stomach. Furthermore, the 10 MV FFF beam, which is produced by removing a flattening filter in the beam path of a conventional 10 MV FF beam, was used for VMAT to reduce the beam delivery time with a 4-fold increase in beam intensity. The photon energy of the 10 MV FFF beam is slightly lower than that of the 10 MV FF beam. Compared to the CK_original_ plans with the 6 MV FFF beam, the VMAT plans with 10 MV FFF shows small hot spots (clV_D<10Gy_ in Table 4), which could be attributed to the use of higher beam energy in VMAT than CK.

The beam arrangement in VMAT plans is another issue to be discussed. Although the incorporation of non-coplanar beams has improved normal tissue sparing in liver IMRT studies, the typical clinical VMAT liver plans are still coplanar, and consist of a pair of (clockwise and counterclockwise) arcs, using collimators with a 90° rotational offset. To achieve dose conformity and liver sparing, manually selected non-coplanar VMAT has been clinically used, but its benefit has not been quantified. A recent study [30] compared coplanar VMAT (cVMAT) vs non-coplanar VMAT (nVMAT) plans in liver SBRT, still presented controversial results; normal liver volume receiving > 15 Gy reduced 5 cc in non-coplanar VMAT than coplanar VMAT, however normal tissue complication probability (NTCP) was 1% lower in cVMAT than nVMAT.

Nevertheless, it should be considered that the dosimetric characteristics of CK plans can be strongly affected by the number of beams and collimators, the sizes of the collimator, and imposed dose constraints on the tumor and OAR during sequential optimization. In the present study, we aimed to achieve a high level of dose conformity to the tumor as well as dose compactness, with carefully designed dose limiting auto-shells.

In addition, the small sample size of this study cohort may be problematic, particularly with regard to the relatively small GTVs (5.1 ± 5.0 cc); therefore, it’s applicability to larger tumor cases should be assessed. Nonetheless, to rigorously validate the results of statistical analysis in this study we computed a posterior statistical power using PS software [31] for the sample size of 29. As summarized in S1 Table, they all show high statistical power, considering a conventional choice of power to compute sample size is either 80% or 90% [32]. In addition, for illustrative purposes, statistical powers as a function of sample size for the main dosimetric parameters between VMAT and CK_modified_ are shown in S1 Fig.

For precise tumor targeting, fiducial markers implanted around the tumor are generally used for setup verification and tumor motion monitoring. In patients previously treated with transarterial chemoembolization using an embolic agent [33], the embolized area successfully served as a direct surrogate for tumor localization on gated fluoroscopy and CBCT during linac-based VMAT treatment. It might be also used for robot-based liver SBRT with the recent fiducial-less tracking capability of CK for respiratory tumor tracking [21].

As stated earlier, even though SBRT can be effectively delivered using the linac systems with various efficient motion management strategies, CK is ideal for the treatment of HCC as its respiratory tumor tracking capability eliminates the need for the ITV margin [20, 21]. With the CK system, SBRT is delivered in the setting of near real-time tracking of implanted fiducial markers combined with respiratory motion modeling to achieve high level of accuracy by continually detecting and correcting for tumor motion throughout the treatment. In such a case, CK can compensate for inconsistent breathing motion with reduced treatment margins, particularly by compensating for the end-of-exhale baseline shift [34]. Through an analysis of the treatment log files of 44 lung cancer patients, it was found that the average clinical accuracy of the respiratory motion tracking system was within 2.5 mm [35]. A similar analysis conducted in 23 lung cancer patients also showed that the margin expansions needed for 95% target coverage was at most 3.5 mm in the superior–inferior direction [36]. They fit well within the 5 mm PTV margin that is currently used at our institution. Furthermore, the CK Synchrony system has the benefit of a 100% duty cycle, unlike the 30–50% duty cycle of the gated treatments.

In our analysis, we directly compared these two treatment techniques (VMAT with ITV margin vs. CK with or without ITV margin) in terms of plan quality, using several dosimetric indices. Overall, by eliminating the ITV margin of the respiratory-gated treatment, the CK_modified_ plans saved about 60 cc more critical normal liver volume than the VMAT plans, given the mean normal liver volume of 1164 cc in our patient cohort. The relationship between the radiation dose to normal liver tissues and the incidence of hepatic toxicities had previously been studied. Hepatic toxicity such as RILD is one of the important complications of SBRT [10, 13, 14]. Schefter et al. reported that the liver constraint for that regimen required at least 700 cc of normal liver volume (excluding the GTV) receiving < 15 Gy [15], and Liang et al. also reported that the liver volume receiving > 20 Gy (nlV_D >20Gy_) was the most significant dosimetric parameter [37]. The Quantitative Analysis of Normal Tissue Effect in the Clinic (QUANTEC) recommends a mean normal liver dose (excluding the GTV) < 15 Gy for 3 fractions, and < 20 Gy for 6 fractions in HCC patients receiving SBRT, and at least 700 cc of normal liver volume receiving < 15 Gy in 3 or 5 fractions [14]. In our recent study investigating the threshold dose for hepatic parenchymal changes on gadolinium ethoxybenzyl diethylenetriamine pentaacetic acid (Gd-EOB-DTPA)-enhanced MRI images after SBRT for HCC [25], the hepatic parenchymal reaction dose for the parenchymal changes in the hepatobiliary phase of the enhanced MRI images performed 2–4 months after 45 Gy was delivered in 3 fractions, was approximately 20 Gy. Nevertheless, given that the understanding of the normal liver dose constraints was generally derived from retrospective chart reviews or via mathematical modeling, all dose recommendations are associated with some uncertainty and should be interpreted cautiously.

All the plans in the present study were calculated using the actual treatment parameters: the two arcs technique for the VMAT plans, and sequential optimization with fixed collimators for the CK plans. The estimated treatment time for the CK plans was significantly longer (50–60 min) than that for the VMAT plans (approximately 20 min). In the CK plan, hundreds of non-isocentric and non-coplanar cone based radiation beams are delivered to the edge of the target, thus creating a highly conformal dose distribution with a sharp dose fall-off. The disadvantage conferred by the long treatment time with CK, is associated with the use of fixed-sized collimators, and can therefore somewhat be mitigated with the use of IRIS^™^ (variable aperture with 12 dodecagon-shaped collimators of 5–60 mm) or InCise^™^ (multi-leaf collimators with 41 leaf pairs, maximum field 10 × 12 cm). Several feasibility studies have shown that the addition of IRIS (35 min) or MLC (23 min) collimator to the CK system would reduce the treatment time per fraction compared to the fixed collimator (53 min) [38–41].

## Conclusions

The tumor-tracking capability of the CK system can preserve a significantly larger volume of critical normal liver tissue receiving < 15 Gy, when compared to the linac-based VMAT technique with respiratory-gated treatment, while maintaining high precision in target localization, conformity, tumor coverage, and the sparing of the OAR. Thus, it can be a valuable SBRT option, particularly for HCC patients with poor liver function.

## Supporting Information

S1 Fig Statistical powers as a function of the sample size for the assessment of the main dosimetric parameters between VMAT and CK_modified_, which was calculated using PS software [31]. Given the sample size of 29, the powers reached >90%, except for GI_50%_.
(TIF)

S1 Table Main conclusions of this study and relevant statistical powers for the sample size of 29. (DOCX)
