# Supplementary figures and images for: Gated Volumetric-Modulated Arc Therapy vs. Tumor-Tracking CyberKnife Radiotherapy as Stereotactic Body Radiotherapy for Hepatocellular Carcinoma: A Dosimetric Comparison Study Focused on the Impact of Respiratory Motion Managements
Source: PLoS One. 2016 Nov 22;11(11):e0166927. doi: 10.1371/journal.pone.0166927 (PMC5119818; doi:10.1371/journal.pone.0166927)

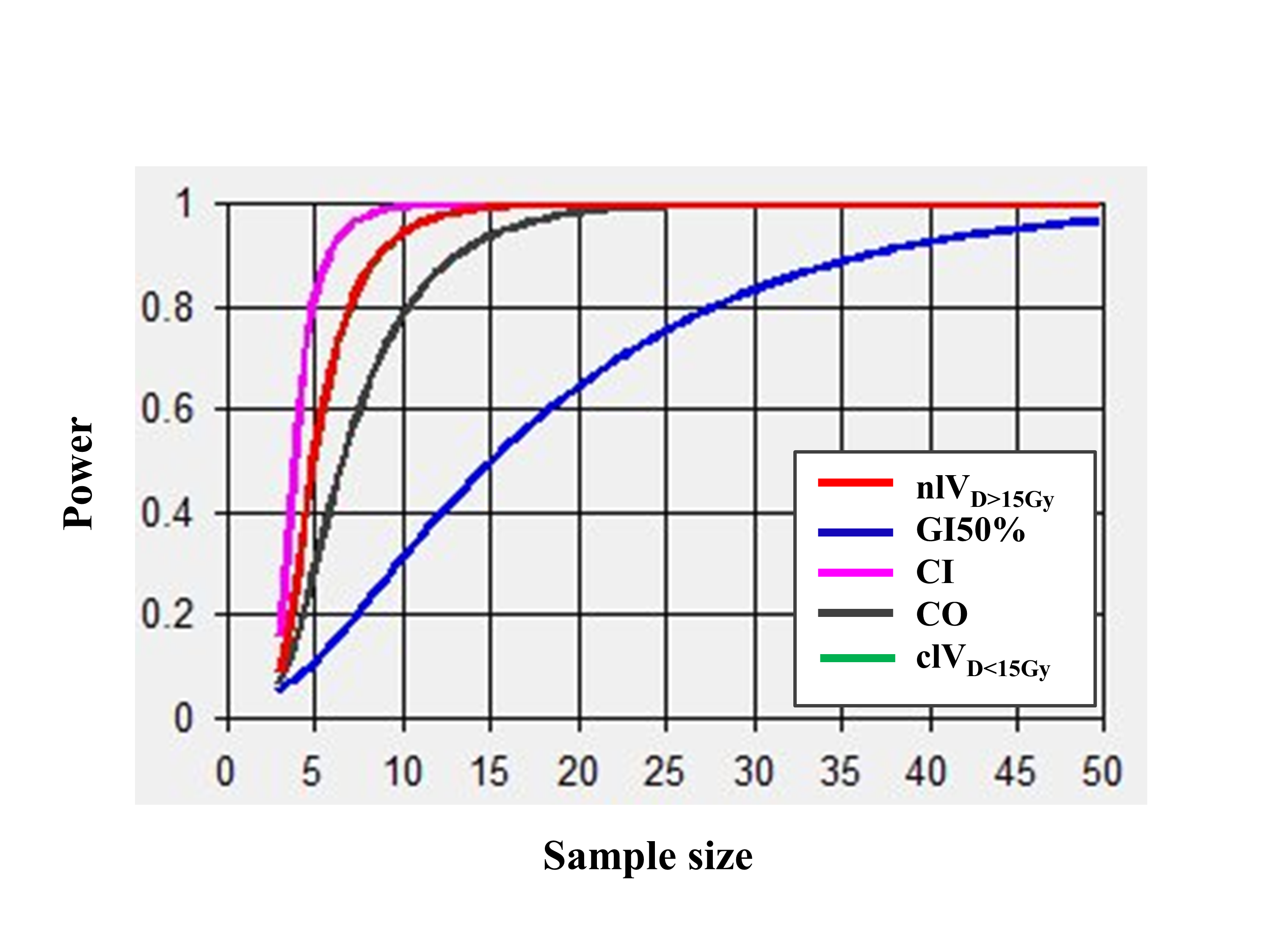

Supplement: S1 Fig — Given the sample size of 29, the powers reached >90%, except for GI50%. (TIF) [file pone.0166927.s001.tif]
