# Supplementary material for: Gated Volumetric-Modulated Arc Therapy vs. Tumor-Tracking CyberKnife Radiotherapy as Stereotactic Body Radiotherapy for Hepatocellular Carcinoma: A Dosimetric Comparison Study Focused on the Impact of Respiratory Motion Managements
Source: PLoS One. 2016 Nov 22;11(11):e0166927. doi: 10.1371/journal.pone.0166927 (PMC5119818; doi:10.1371/journal.pone.0166927)
Supplement: S1 Table — (DOCX) [file pone.0166927.s002.docx]

**S1 Table.** Main conclusions of this study and relevant statistical powers for the sample size of 29.

| Main conclusions | VMAT | CK_modified_ | *p*-value | Power |
| --- | --- | --- | --- | --- |
| nlV_D>15Gy_ | 128.0 ± 71.4 | 73.1 ± 42.6 | < 0.001 | 1.000 |
| High precision in target localization: GI_50%_ | 3.83 ± 0.56 | 3.62 ± 0.40 | < 0.005 | 0.819 |
| Conformity index | 1.05 ± 0.05 | 1.17 ± 0.05 | < 0.001 | 1.000 |
| Tumor coverage | 93.0 ± 3.9 | 96.9 ± 2.5 | < 0.001 | 0.998 |
| Normal liver sparing: clV_D<15Gy_ | 1034.3 ± 205.3 | 1091.2 ± 195.3 | < 0.001 | 1.000 |

Note that the conventional choice of power to compute the sample size is either 80% or 90%.
